# Medical education during the COVID-19 pandemic: What students missed and what they did not. A questionnaire-based cross-sectional study

**DOI:** 10.3205/iprs000175

**Published:** 2023-07-25

**Authors:** Giulia Manzini, Marko Kornmann, Michael Kremer

**Affiliations:** 1 Department of General and Visceral Surgery, Hospital of Aarau, Switzerland; 2 Department of General and Visceral Surgery, University Hospital of Ulm, Germany; 3 Department of Surgery, Hospital of Lachen, Switzerland

**Keywords:** medical education, strategies, online learning, pandemic, skills lab

## Abstract

**Background:**

Medical education was and still is challenged by the COVID-19 pandemic, and several strategies were implemented by the universities worldwide in order to maintain a good level of education. The aim of this work is to point out how strategies adopted in a German university hospital reached students and how comfortable they felt with the proposed solutions in order to define future possibilities in modern teaching.

**Methods:**

A questionnaire was answered by medical students at the end of the 8^th^ and 10^th^ semester in a German university hospital asking them about their perception of medical education during the pandemic as well as about strategies adopted by the faculty.

**Results:**

A total of 92 out of 117 students answered the questionnaire (78.6% response rate). Students felt disadvantaged in their medical education because of the pandemic on a scale from 0 (not at all) to 10 (completely) (5.34±2.3, range 0–10 points), regardless of semester, gender, and whether they aimed at a surgical career or not. During the pandemic they missed practical exercises most (93.5%), followed by contact with other students (65.2%). Presence lessons were missed (28.3%) the least. Among the strategies offered to maintain education, recorded lessons were appreciated most, followed by skills labs. Live-stream lessons were considered less comfortable.

**Conclusions:**

Several aspects of medical education were replaced satisfactorily during the pandemic, others need to be adapted in the future in order to meet the students’ needs and expectations. Theoretical online education but not live stream lessons could be an option beyond COVID-19 as they are highly appreciated by students.

## Zusammenfassung

**Einleitung:**

Die medizinische Ausbildung wurde durch die COVID-19-Pandemie massiv herausgefordert. Die Universitäten weltweit entwickelten und setzten mehrere Strategien ein, um ein gutes Ausbildungsniveau aufrechtzuerhalten. Ziel dieser Arbeit ist zu untersuchen, wie die adoptierten Strategien von den Studenten aufgenommen wurden, um die medizinische Ausbildung zukünftig anzupassen.

**Methode:**

In einem deutschen Universitätsklinikum erhielten 117 Medizinstudenten im 8. und 10. Semester einen Fragebogen mit mehreren Fragen über ihre Beurteilung der Qualität der medizinischen Ausbildung während der Pandemie sowie über die Effektivität der durch die Fakultät ergriffenen Maßnahmen, um diese aufrecht zu halten. Werte wurden als Mittelwert [± Standardabweichung] und Medianwert [Bereich] für kontinuierlichen Variablen angegeben. Dichotome Variablen wurden sowohl als absolute Zahl als auch als Prozent dargestellt. Die Gruppen wurden mit dem Student-t-Test für unabhängige Stichproben verglichen.

**Ergebnisse:**

92 von 117 Medizinstudenten (78.6%) beantworteten den Fragebogen. Auf einer Skala von 0 (überhaupt nicht) bis 10 (total) fühlten sich Studenten 5,34±2,3 (Bereich 0–10 Punkte) aufgrund der Pandemie in ihrer medizinischen Ausbildung benachteiligt, unabhängig von Semester, Geschlecht und ob sie eine chirurgische Laufbahn anstrebten oder nicht. Während der Pandemie vermissten sie vor allem praktische Übungen (93,5%), gefolgt vom Kontakt mit den Kommilitonen (65,2%). Präsenzvorlesung wurde am wenigstens vermisst (28,3%). Unter den angebotenen Strategien zur Aufrechterhaltung der medizinischen Ausbildung wurden online aufgezeichnete Lektionen am meisten geschätzt (8,2/10 Punkte), gefolgt von Skills Labs (7,44/10 Punkte). Live-Stream-Unterricht wurde als weniger komfortabel bewertet (6,6/10 Punkte).

**Diskussion:**

Einige Aspekte der medizinischen Ausbildung wurden während der Pandemie zufriedenstellend ersetzt, andere müssen in Zukunft angepasst werden, um den Bedürfnissen und Erwartungen der Studenten gerecht zu werden, wie z.B. mehr Tutorials, die klinische Untersuchungstechniken zeigen, und weniger Live-Stream-Unterricht. Online aufgezeichnete Vorlesungen könnten eine Option über COVID-19 hinaus sein, da diese von den Studenten sehr geschätzt wurden.

## Introduction

The pandemic affected medical education worldwide extremely [1], disrupted routines in hospitals and medical schools [2], mainly where practical activities were necessary. In order to contain the infection, internships in the different wards were cancelled or shortened considerably, bedside teaching was suspended and frontal lessons were replaced by online seminars [3]. Reasons for cancelling clinical clerkships were, firstly, to flatten the curve, with the goal of minimizing personal interactions to mitigate and contain the spread of COVID-19, secondly, to decrease the risk of exposure for medical students, and, finally, to save protective equipment in order to ensure that healthcare workers were able to protect themselves adequately [2]. The cancellation of clerkships, which are necessary for both skill acquisition as well as for relationship building, is a serious issue which students and medical universities had to solve [2]. Online platforms were established aiming to maintain the educational process [4], [3], [5]. 

In order to learn from this pandemic and to be prepared eventually if a new one were to occur, it is not only important to evaluate the impact of the COVID-19 pandemic on medical education or examinations, as several studies have done already [1], [3], [6], [7], but also to analyze how students dealt with this new situation and with the new learning and teaching methods as well as what their personal perception was in order to be sure that resources will be used in the right way in the future. In this regard, our objective is to examine the perception of medical students of the changes applied to medical education during the COVID-19 pandemic with a questionnaire-based study.

## Methods

### Questionnaire

**Figure 1:**
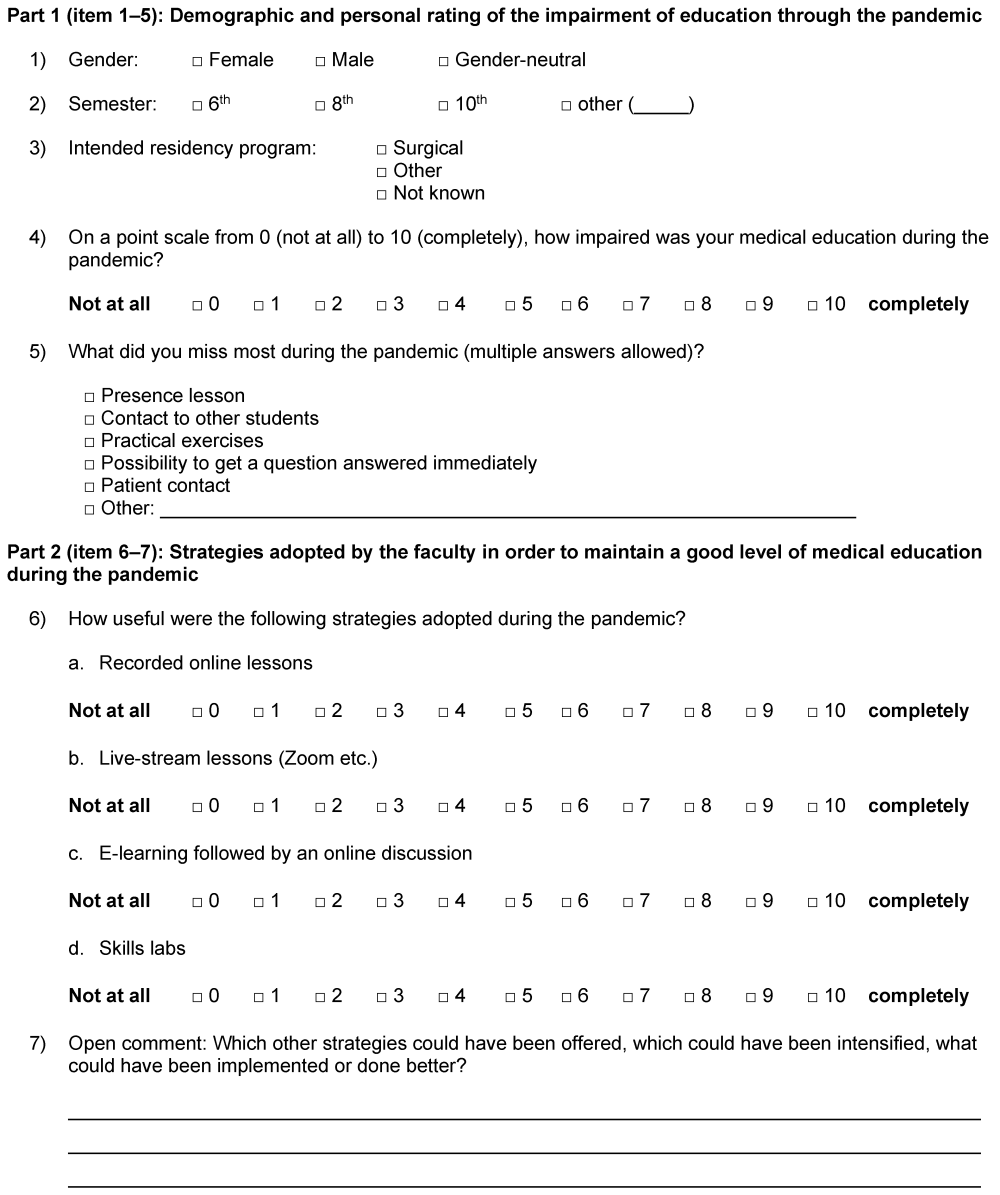
Seven-item questionnaire

This was a questionnaire-based cross-sectional study that followed a surgical OSCE (objective structured clinical examination) for students at the end of the 8^th^ or 10^th^ semester of medical education in order to explore their perceptions of the pandemic time at the University of Ulm, Germany. In order to evaluate the students’ perception of medical education during the pandemic, a 7-item questionnaire (Figure 1 (Fig. 1)) was developed by two senior surgeons (GM and MKr) routinely involved in the examination of medical students. In the first part (items 1–5), following three questions about gender, attending semester, and intended residency program, students were asked to give their personal rating of the impairment of education due to the pandemic as well as what they missed most during the pandemic. In the second part (items 6–7), students had to evaluate the strategies adopted by the faculty in order to maintain a good level of medical education. The last question (item 7) was an open question. The structure and content validity of the questionnaire were checked by two faculty members (MKr and DHB). All students (n=117) who participated in the OSCE examination in winter semester 2022/2023 were invited to participate in this survey directly after the end of the OSCE assessment. No personal identifiers were collected, and the study adhered to the ethical principles of the Helsinki declaration. 

### Statistical analysis

Values are presented as mean [± standard deviation] and median [range] for continuous variables. Dichotomic variables are presented as absolute numbers (frequency) as well as percent. Groups were compared with the student t-test for independent samples. A two-sided p-value <0.05 was considered statistically significant. Missing values were <5% in the data set, and no imputation strategies were needed. All calculations were conducted using R Project for Statistical Computing (The R Foundation, Version 3.1.0, Vienna, Austria).

## Results

117 students were invited to participate in the survey, and 92 students filled out the first part of the questionnaire at the end of the OSCE examination (78.6% response rate). Of those participants, 84 (91.3%) also filled out the second part of the questionnaire about strategies adopted by the faculty to maintain a good level of education. The majority of the participants were female (80.4%) and at the end of the 8^th^ semester (72.7%). Only 30 students (32.6%) were oriented for a surgical residency program after completing the 6-year medical formation (Table 1 (Tab. 1)). 

Students felt themselves mildly disadvantaged in their medical education during the pandemic (5.34±2.3, range 0–10 points). No difference was observed in this perception regarding whether the student intended to absolve a surgical career (n=30) or not (n=47), with scores of 5.06±2.5 vs. 5.57±2.4 (p=n.s.), respectively. The 15 students who still did not know which specialty is of interest to them had a slightly higher score (5.7±1.38). No difference was observed regarding the attended semester (5.84±2.44 for students in the 8^th^ semester and 5.15±2.23 for students in the 10^th^ semester, p=n.s.) or gender (4.5±2.33 for male vs. 5.5±2.26 for female, p=n.s.). As shown in Table 2 (Tab. 2), students missed practical exercises (93.5%) most during the pandemic, followed by contact with other students (65.2%) and with patients (58.7%). Presence lessons were missed (28.3%) the least. Eighty-four students completed the entire questionnaire evaluating the strategies offered by the faculty to maintain education by a point scale from 0 (not useful at all) to 10 (extremely useful). Recorded lessons were mostly appreciated (8.2±2.6, range 0–10 points), followed by skills labs (7.44±3.4, range 0–10 points). Live-stream lessons (6.6±2.6, range 0–10 points) as well as e-learning (6.8±3.4, range 0–10 points) were considered as less useful. Twelve students out of 27 (44.5%) wrote an additional comment wishing for more skills labs and practical exercises, also in form of online tutorials/videos.

## Discussion

The pandemic challenged the global educational and healthcare systems enormously [8]. In this context virtual education moved into the central position to maintain medical education during the COVID-19 pandemic [8]. Online lessons, live-stream seminars, and online platforms were provided in order to supply the lack of face-to-face lessons [4], [5]. Generally, students felt mildly disadvantaged in their medical education during the pandemic, regardless of age, gender, and attended semester. This study demonstrated, on the one hand, that students felt comfortable with online seminars, less with live-stream lessons. This was probably due to the greater flexibility offered by recorded seminars, as students were free to watch them whenever they wanted and had time for it. On the other hand, the biggest problem during the pandemic was the lack of practical exercise and the contact with other students. As emerged previously in a paper published by our group [9], during the pandemic students performed worse on the surgical OSCE at the stations where a complete abdominal examination was needed. This finding is reflected in the present results of the questionnaire, as students wished for more practical exercise during the pandemic. This indicates that it is extremely difficult and challenging to replace patient contact and practical learning, and this should be the goal for the future. 

Should a new pandemic occur, video tutorials on examination techniques should be proposed to promote practical skills. In contrast, live-stream seminars, which are surely also more time intensive for faculty members than recorded ones, can be reduced or even abolished, as they were not much appreciated by the students. While lectures can be delivered easily online, surgical skills training, which requires a high level of teacher-student interaction, may be challenging to demonstrate via pre-recorded videos [10]. New models could be offered to the medical students as, for example, the online web-based interactive surgical training described by Co et al. [11]. In this case-control study surgical skills performance was compared between students who were taught through the web-based interactive surgical training and those who were taught in a conventional face-to-face tutorial. A meta-analysis published in 2022 by Mao et al. found that basic surgical skills could be taught as effectively through online video-based education as through conventional teaching methods [12]. These results are consistent with previous data published by our group, demonstrating that suturing on skin models was even better during the pandemic [9]. Another discussion point is whether recorded lessons, which were highly appreciated by students, could be routinely used in specific contexts throughout the medical curriculum, even after the end of the pandemic. In their meta-analysis Mao et al. found that online education should be utilized as an adjunct to medical curricula beyond the COVID-19 era [12]. This would allow students to organize their time more effectively, even, for example, by spending more time in medical and surgical wards than required by the curriculum, rather than being concentrated with too many students in the “official” clerkships. This would probably help students to better identify what specialty they really like. The choice is difficult, and in our questionnaire 16% of the students still did not know which specialty they would like to pursue even towards the end of the medical school. Online formats are generally perceived as good options, as also described in the questionnaire-based study by Shaiba et al. [8]. Here, students and faculty were invited to give their opinion on an electronic objective structured clinical examination (e-OSCE) that was designed for the final-year medical students at a university in Saudi Arabia. The examination was administered by Zoom^TM^ video conferencing. At the end of the examination an online questionnaire was undertaken with both students and faculty in order to explore their perception of this experience. The response rate was 54% (73 out of 136 participants). 69.8% were very comfortable with the new virtual experience. 53.4% preferred the e-OSCE compared to the classic face-to-face clinical OSCE. However, one limitation of this study is the low response rate, which could result in a large bias, as the 46% who did not complete the online questionnaire probably did not feel comfortable with the format. 

Katmeh et al. [7] also performed a questionnaire-based study on 46 3^rd^ year medical students at the St. George’s University of London. The response rate was also very low here, i.e. 22% (206 students received the shared link to participate). The questionnaire included two sections to elicit ideas on both the current and future implications of COVID-19 on 3^rd^ year examination and future clinical placements. 63% of the students performed better in the clinical OSCE compared to the written exam, suggesting that they felt disadvantaged by the cancellation of the OSCE. The greatest difficulty encountered by students conducting the online examination was the home environment. A lack of clinical skills was the greatest concern of the students. Similar to our results, the importance of bedside teaching with a real patient also emerged here. It has to be underlined that the response rate was only 22%, as a total of 206 students received the questionnaire, raising the question why so many students did not answer and if the results have enough internal and external validity to be representative of third year medical students. The response rate of our questionnaire was 78.6%, probably as it was administered after a face-to-face OSCE examination, and students found the sheet on their way out of the examination room together with a questionnaire regarding their perception of the examination itself.

## Conclusions

In conclusion, online lessons could also be a good option for medical education after the end of pandemic in some fields; live-stream seminars are not as much appreciated. If clerkship cannot be offered to students during the pandemic, more videos with practical online tutorials should be offered as well the opportunity to train regularly in a skills lab. In particular, clinical examination techniques should be demonstrated as well as models to train basic surgical skills beyond skin suturing.

## Notes

### Ethics statement

 The study adhered to the ethical principles of the Helsinki declaration. 

### Availability of data and materials

The datasets generated and analyzed for the current study are available from the corresponding author on reasonable request.

### Authors’ contributions

GM and MKr wrote the manuscript; GM did the statistics; MKr and GM did the tables; MKo was responsible for the questionnaire. All authors revised the manuscript and approved the final version.

### Acknowledgements

All authors thank Professor Doris Henne-Bruns for reviewing the content of the questionnaire.

### Author’s ORCID

- Giulia Manzini: 0000-0002-8032-8043

### Competing interests

The authors declare that they have no competing interests.

## Figures and Tables

**Table 1:**
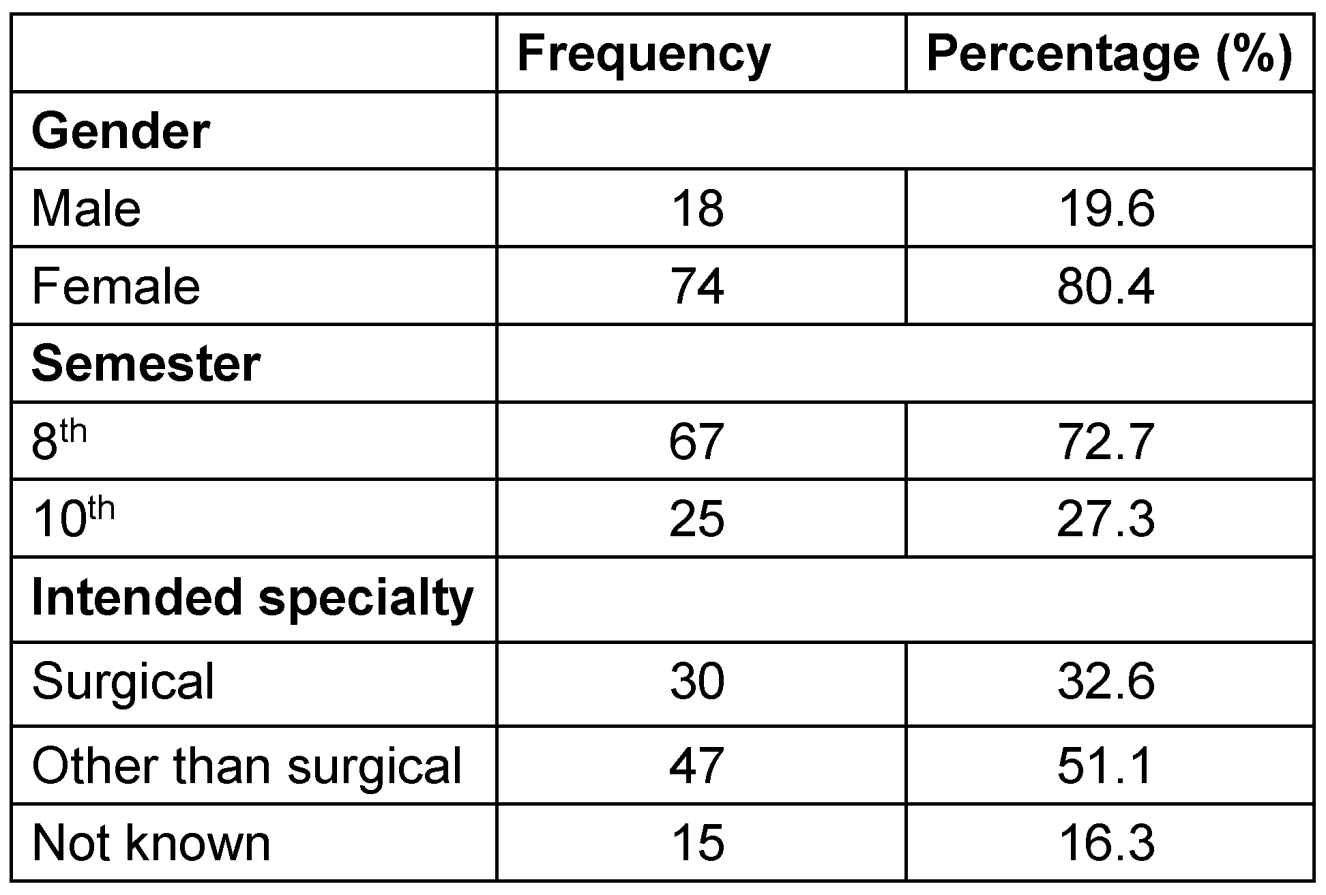
The characteristics of the students participating in the survey (n=92)

**Table 2:**
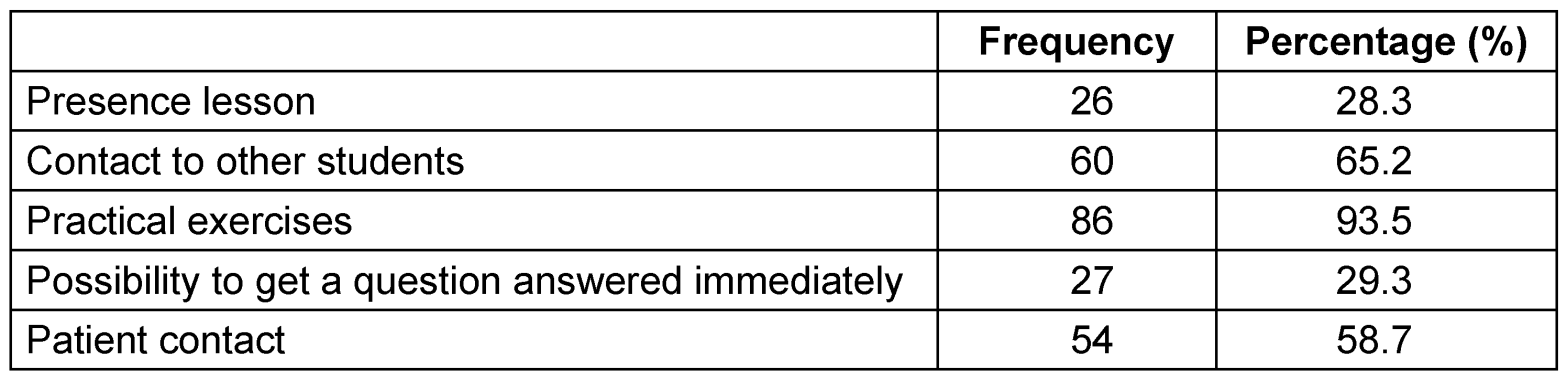
What students missed (n=92)
